# Indian Journal of Orthopaedics: The journey so far

**DOI:** 10.4103/0019-5413.58599

**Published:** 2010

**Authors:** Anil K Jain, DK Sahu

**Affiliations:** Editor, Indian Journal of Orthopaedics, and Professor Orthopaedics, UCMS, Delhi, India; 1 Editor, Indian Journal of Medical Sciences, and Managing Director, Medknow Publications and Media Pvt Ltd, Mumbai, India

Science is ever changing. For the growth of science, one needs to have a problem-based solution. The path to solve a problem starts with identification of the cause. Once the cause is identified strategies can be planned to solve the problem. The process of finding the cause and the solution forms the basis of a research design and scientific study. Scientific evaluation of outcome with a longitudinal collection of data which stands the test of valid statistical analysis becomes definite evidence. The study design, if reproducible in the hands of other clinicians and researchers, could influence the practice of medicine. Such clinically relevant evidence once generated should be available beyond the geographic boundaries and human lifespan. Such widely accessible and retrievable evidence contributes to the growth of science and that is how the clinical practice is changed. Results of good research which are available globally and beyond human lifespan have allowed modern medicine to advance. Peer reviewed scientific journals play a big role in dissemination of results from research. Such journals not only allow documentary evidences for the present and future generations but also provide a forum for scientific scrutiny of the research papers by the peers.

The Indian Journal of Orthopaedics (IJO) was started well before Orthopaedics was a well established specialty in India. The Indian Orthopaedics Association (IOA) was then a section of the Association of Surgeons of India. IJO was started in 1967 with two issues per year.A visionary step to document the research in the country, it was also a herculean task to attract quality manuscripts, perform the peer review and take care of the publishing process in an era when computers and internet were non-existent. In spite of these difficulties, the quality of IJO was consistently maintained. In the year 1994, the frequency of the IJO was increased to four issues per year. Although we were consistent with the publication schedule, the circulation of the journal was only to the members of Indian Orthopedic Association and hence the readership was limited to the members. The articles published in the journal were not cited very often. The journal was not indexed with any major Abstracting and Indexing (A& I) database. Hence, manuscripts submitted to and published by IJO were very low in numbers.

The current editorial team, which took the charge in the year 2007, was given one year to pen the issues related with IJO and formulate a plan to overcome the shortcomings and difficulties. During the planning phase, a lot of thought process went into increasing the visibility, maintaining the consistency of quality, objective documentation of the peer review process, requirement for indexing with international A& I databases, need for a professional publisher and the finances involved.

## GLOBAL REACH

**Figure 1:**
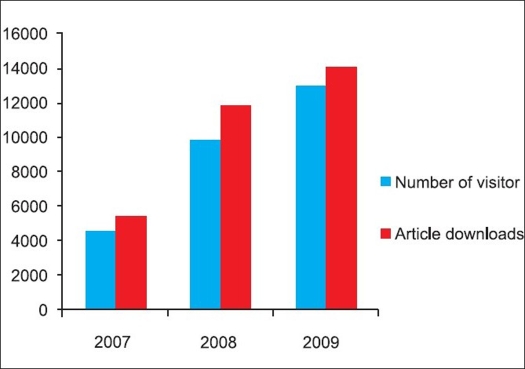
Monthly average visitors and article downloads from www.ijoonline.com [Source: Google Analytics]

**Figure 2:**
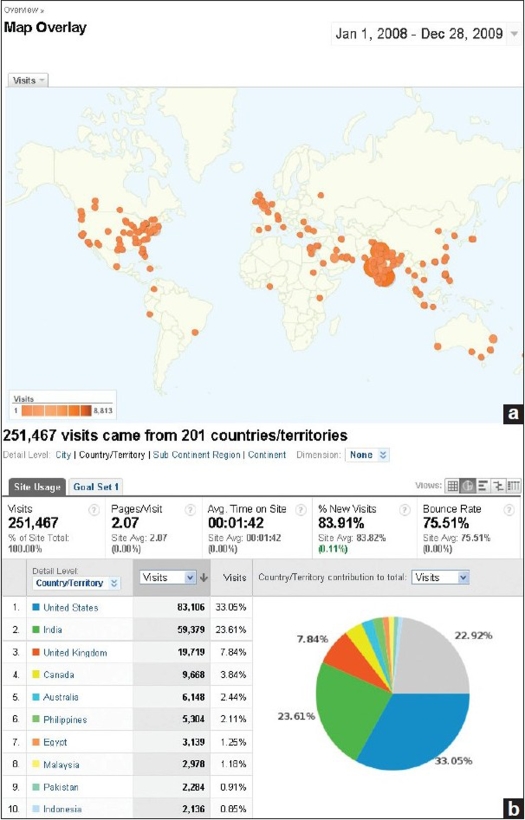
Geographic distribution of visitors on www.ijoonline.com from the year 2008 onwards [Source: Google Analytics] (a) City-wise distribution (b) Country-wise distribution

**Figure 3:**
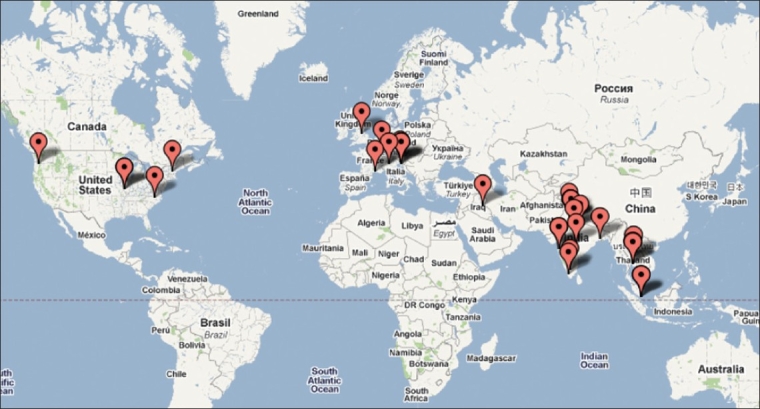
Author institution mapping for the this issue of the Indian Journal of Orthopaedics

**Figure 4:**
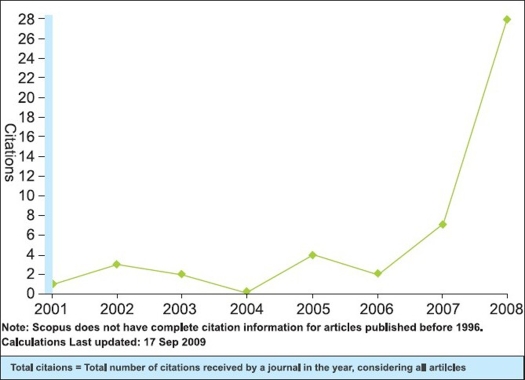
Citation graph showing increase in citations being received for the Indian Journal of Orthopaedics (Source: SCOPUS)

Good work published should be easily retrievable by other researchers so that more work can be built on that and the results can be utilized in clinical practice. With the aim to increase visibility and ‘research impact,’ we launched a separate website for the IJO with the domain www.ijooneline.com during the IOACON 2006 at New Delhi. The website offers immediate unrestricted free access to all the content of the journal. In fact, the journal now publishes most of the articles within a few weeks of acceptance as ‘Ahead of Print’ after completing the entire pre-publishing process of technical and language editing. The back issues up to the year 2002 have also been made available online with free access. The website of the journal provides a number of user friendly features such as reference linking, article and issue level metrics such as downloads and citations, citation alerts for the authors, author institution mapping, etc. The open access policy of the journal, has led to increase in the visibility of its content globally which is reflected by number of visitors and article downloads from its website. The increased visibility of the journal led to increase in the manuscripts submitted and citations received for the published articles [Figures 1–4].

## ONLINE SUBMISSION SYSTEM AND PEER REVIEW PROCESS

**Figure 5:**
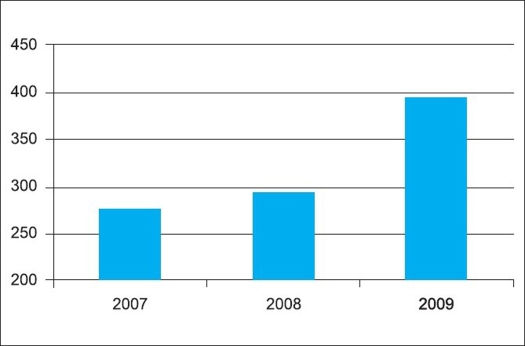
Number of manuscripts submitted to the Indian Journal of Orthopaedics (2007-2009)

**Figure 6:**
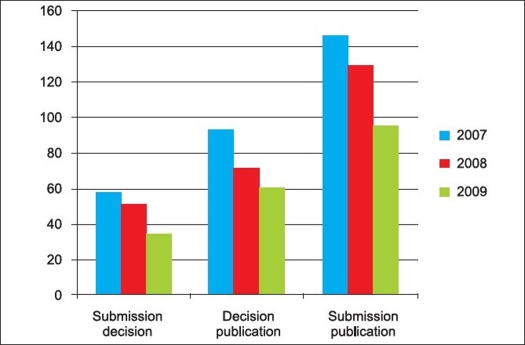
Review timeline of the Indian Journal of Orthopaedics (2007-2009)

**Figure 7:**
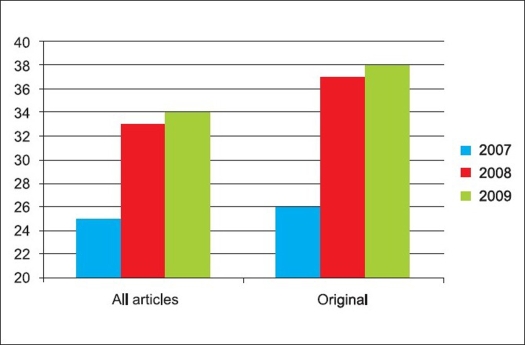
Contributions from outside India to the Indian Journal of Orthopaedics (2007-2009)

The entire review process of the journal is managed through a web-based manuscript processing system (www.journalonweb.com/ortho). The system efficiently manages the double blind peer review of manuscripts and has led to decrease in submission to decision timeline. After the initial review of the manuscript by one of the editorial board members, it is sent to three to five subject experts, who are either selected from the existing database of the journal or after a search from other A and I databases. The final decision on the manuscript is taken by the editor based on the available comments from the peers. The system allows authors to verify the style and accuracy of references after cross-checking the bibliographic elements from PubMed. The editorial team can check for possibility of plagiarism or duplicate system. The authors are notified by a mobile message when the manuscript is sent for revision or proofs. Better management of the process had led to increased confidence of the authors and increase in submission of manuscripts from India as well as from other countries. The journal is now able to ‘choose’ better quality articles as can be seen from the increasing rejection rate from less than 60% in 2007 to over 75% in 2009 [Figures 5–7].

## PRE-PUBLICATION PROCESS

Apart from review of the manuscripts, the most tedious and time consuming aspect in the entire publishing process of a journal is the pre-publication process of editing and proofing. Technical and scientific editing of accepted manuscripts takes time and requires expertise. Good technical editing does improve readability and quality of manuscripts. IJO now does all editing and proofing before formally accepting a manuscript. When a manuscript is ready for acceptance, all the references cited by the authors are cross-checked and corrected, the manuscript is looked for uniformity of style and the language is corrected. The editorial team then looks into the scientific aspect of the manuscript before it is paginated and shown to the authors. After this it is published online as ‘Ahead of Print’. The editorial team along with publisher worked on the overall design and layout of the journal to make it appealing and easy to read.

## THEME-BASED ISSUES

The editorial team decided to bring two to three issues every year on specific topic. We included a symposium in each issue on a topic of clinical relevance and involved renowned experts on the subjects. Each such issue has two to three state-of-art reviews, 8-10 research papers and an editorial highlighting the consensus achieved on the subject of symposium and enumerating directions for the future research. In the last three years we have published theme-based issues on research methodology and evidence based medicine, giant cell tumor, spinal trauma, fracture neck of femur, total hip arthroplasty, open fracture, avascular necrosis of hip and bone stimulation.

## STRENGTHENING OF THE EDITORIAL BOARD

Till now the IJO had an elected editorial board. Recently the IOA has brought a constitutional amendment to select the editors and editorial board members based on fixed criteria including academic merit and publications. The so called ‘thank-less’ job of editors, which creates more ‘enemies’ than friends, requires skills which can be learnt by those who have love for the job and academics. Acquiring editorial skills takes time and hence it is necessary to groom new editorial board members and future editors. This step of having merit-based selection of editorial board members would definitely help in maintaining and improving the quality of the journal. Similarly, to give international fervor to the editorial board, a number of distinguished personalities were invited to be part of it and to its credit majority willingly joined IJO's board.

## WHERE DO WE STAND TODAY?

This ‘all out’ approach towards overhauling the journal has paid rich dividends. A good journal needs good quality manuscripts, support of dedicated reviewers and readers. IJO now has this base of good articles, both in quantity and quality, reviewers and readers, as can be seen from increasing number of new submissions and downloads and citations of published articles. The journal is now indexed with PubMed and archived with PubMed Central. IJO has also been included in Science Citation Index Expanded from January 2008 and we shall have our first Impact Factor in June 2011. The inclusion in PubMed and Science Citation Index will further increase the journal's visibility. This should encourage all the authors in this subcontinent and outside to submit their research work to IJO.

## WHERE DO WE GO FROM HERE?

We still have a long journey towards making IJO a journal which can make impact on clinical practice and academics. IJO has to first become one of the most sought after journal, both by readers and authors. Though we understand the limitations of Impact Factor and its use in judging quality of research and a journal, we need to improve on our citations and Impact Factor so that authors from universities and institutions requiring publication in high IF journals as criterion for promotion can consider IJO as an alternative to other closed access but high-IF journals. We would give high importance to studies focusing on the problems of the developing world. To ensure that, we have planned to digitize all our back issues from the inception of IJO and upload these on the journal's website. These digitized issues would also be archived at other repositories such as Portico and through CLOCKSS.

We are thankful to our authors, reviewers, editorial board members, all the staff at Medknow Publications and media Pvt. Ltd. and members and office bearers of IOA and hope to continue working together in this journey. We believe, together we can and together we shall attain new heights!

